# The First Berberine-Based Inhibitors of Tyrosyl-DNA Phosphodiesterase 1 (Tdp1), an Important DNA Repair Enzyme

**DOI:** 10.3390/ijms21197162

**Published:** 2020-09-28

**Authors:** Elizaveta D. Gladkova, Ivan V. Nechepurenko, Roman A. Bredikhin, Arina A. Chepanova, Alexandra L. Zakharenko, Olga A. Luzina, Ekaterina S. Ilina, Nadezhda S. Dyrkheeva, Evgeniya M. Mamontova, Rashid O. Anarbaev, Jóhannes Reynisson, Konstantin P. Volcho, Nariman F. Salakhutdinov, Olga I. Lavrik

**Affiliations:** 1 N. N. Vorozhtsov Novosibirsk Institute of Organic Chemistry, Siberian Branch of the Russian Academy of Sciences, 9, Akademika Lavrentieva Ave., Novosibirsk 630090, Russia; liza95@nioch.nsc.ru (E.D.G.); niv@nioch.nsc.ru (I.V.N.); bred@nioch.nsc.ru (R.A.B.); luzina@nioch.nsc.ru (O.A.L.); anvar@nioch.nsc.ru (N.F.S.); 2 Novosibirsk State University, Pirogova str. 1, Novosibirsk 630090, Russia; anarbaev@niboch.nsc.ru (R.O.A.); lavrik@niboch.nsc.ru (O.I.L.); 3 Novosibirsk Institute of Chemical Biology and Fundamental Medicine, Siberian Branch of the Russian Academy of Sciences, 8, Akademika Lavrentieva Ave., Novosibirsk 630090, Russia; arinachepanova@mail.ru (A.A.C.); sashaz@niboch.nsc.ru (A.L.Z.); katya.plekhanova@gmail.com (E.S.I.); elpida80@mail.ru (N.S.D.); evgeniya.mm.94@gmail.com (E.M.M.); 4 School of Pharmacy and Bioengineering, Keele University, Hornbeam Building, Staffordshire ST5 5BG, UK; j.reynisson@keele.ac.uk

**Keywords:** berberine, tetrahydroberberine, Tdp1 inhibitor, cancer, molecular modeling, DNA repair enzyme, SAR

## Abstract

A series of berberine and tetrahydroberberine sulfonate derivatives were prepared and tested against the tyrosyl-DNA phosphodiesterase 1 (Tdp1) DNA-repair enzyme. The berberine derivatives inhibit the Tdp1 enzyme in the low micromolar range; this is the first reported berberine based Tdp1 inhibitor. A structure–activity relationship analysis revealed the importance of bromine substitution in the 12-position on the tetrahydroberberine scaffold. Furthermore, it was shown that the addition of a sulfonate group containing a polyfluoroaromatic moiety at position 9 leads to increased potency, while most of the derivatives containing an alkyl fragment at the same position were not active. According to the molecular modeling, the bromine atom in position 12 forms a hydrogen bond to histidine 493, a key catalytic residue. The cytotoxic effect of topotecan, a clinically important topoisomerase 1 inhibitor, was doubled in the cervical cancer HeLa cell line by derivatives 11g and 12g; both displayed low toxicity without topotecan. Derivatives 11g and 12g can therefore be used for further development to sensitize the action of clinically relevant Topo1 inhibitors.

## 1. Introduction

**Figure 1:**
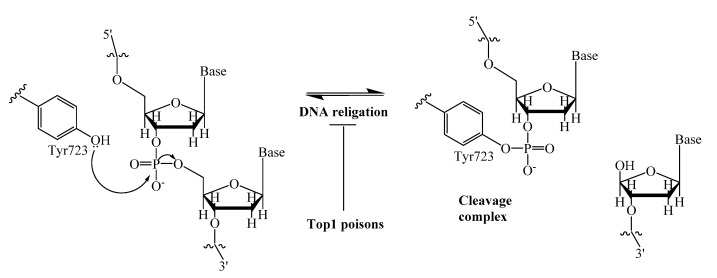
DNA single-strand cleavage by nucleophilic attack of Tyr723 of Top1, and covalent cleavage complex formation.

A promising strategy to enhance the efficacy of anticancer therapy is the inhibition of various DNA repair enzymes, which counteract the effect of many anticancer drugs [1,2]. This is particularly important where resistance to chemotherapy is observed. An interesting example is the poly (ADP-ribose) polymerase (PARP), which inhibitors were studied both in combination with chemotherapeutic agents and as individual drugs. Now olaparib, rucaparib and niraparib are in clinical use for the treatment of ovarian cancers [3]. Another DNA repair enzyme, tyrosyl-DNA phosphodiesterase 1 (Tdp1) has attracted considerable interest in the last few years mainly due to its ability to repair DNA lesions caused topoisomerase 1 (Top1) poisons, a well-established class of anticancer drugs [4]. The anticancer activity of Top1 poisons, camptothecin and its clinically important derivatives, topotecan and irinotecan [5,6], is based on their ability to bind to the covalent intermediate complex Top1/DNA and prevent the restoring of DNA integrity. This leads to the stabilization of the covalent bond between catalytic tyrosine (Y723) of Top1 and the 3′- end of DNA (Figure 1). The Tdp1 mechanism of action is the phosphotyrosyl bond hydrolysis [7], resulting in the resumption of DNA replication and cell division. 

**Figure 2:**
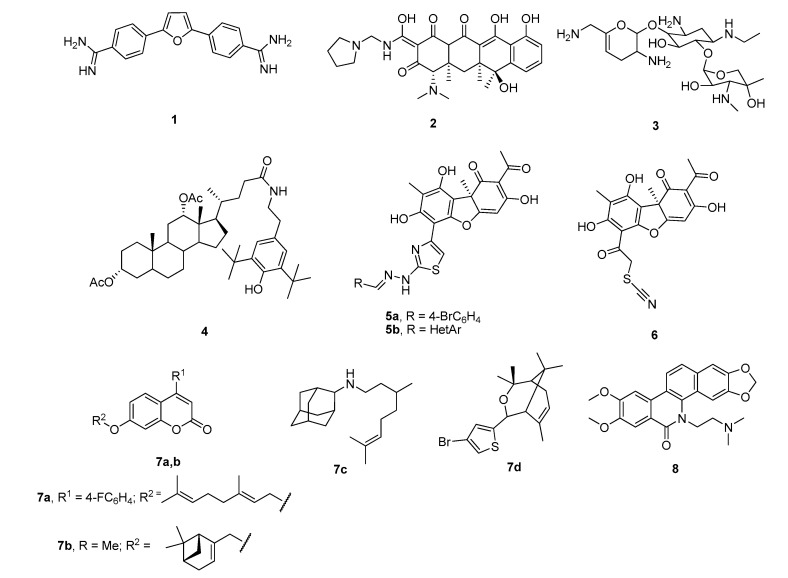
Examples of established Tdp1 inhibitors **1**–**8**.

A few classes of Tdp1 inhibitors are known such as furamidines (compound 1, Figure 2), tetracyclines (compound **2**), aminoglycosides (compound **3**) [8,9]. Also, natural products of various types have been found to inhibit Tdp1, including derivatives of bile acids **4** [10,11], of lichen metabolite usnic acid **5a**,**b** [12,13] and **6** [14], monoterpenoid derivatives **7** [15,16,17,18] and oxinitidine **8** [19] with inhibitory activity in the micro- or submicromolar range. Importantly, the hydrazinothiazole derivative of usnic acid **5a** [13,20] and monoterpene-substituted 4-arylcoumarin **7a** [21] significantly increased topotecan efficacy in vivo.

**Scheme 1:**
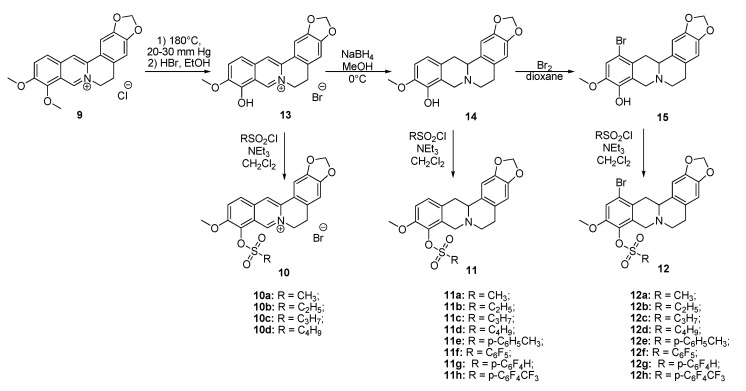
The synthetic pathways to sulfonates **10**, **11** and **12**.

The aim of this study was to establish the potency of a novel structural class of natural products, the derivatives of berberine **9** (Scheme 1), which like their usnic acid counterparts are phenolic compounds. It is known that the isoquinoline plant alkaloid berberines have many beneficial physiological effects, e.g., they are hypocholesterolemic, antibacterial, hypoglycemic agents, antioxidants and, finally, they suppress tumor growth [22,23,24,25,26]. Interestingly, the sulfonate derivatives of berberines and tetrahydroberberines have been reported as promising hypocholesterolemic agents [27,28]. Although berberine derivatives never used as Tdp1 inhibitors, a preliminary molecular modeling study indicated that berberines with 9-sulfonate group would bind to Tdp1. In the present study, 9-sulfonate-berberine and tetrahydroberberine derivatives **10**–**12** with aliphatic and aromatic substitutes were synthesized and their potency against Tdp1 was tested. To the best of our knowledge, this has not been done previously. Using the HeLa cervical cancer cell line, derivatives **11g** and **12g** were found to be nontoxic and sensitized the cancer cells to topotecan. 

## 2. Results and Discussion

### 2.1. Chemistry

Sulfonates **10**–**12** were synthesized in accordance with previously reported methods [27]. For this purpose, berberine **9** was selectively demethylated at 190 °C under vacuum as previously described [29]. After treatment with HBr, berberrubine hydrobromide **13** was isolated in 89% yield (Scheme 1). Compound **13** was reduced with sodium borohydride in methanol according to a procedure described previously [30] yielding tetrahydroberberrubine **14**. The bromination of compound **14** with a bromine in dioxane solution afforded 12-bromotetrahydroberberrubine **15** in 52% yield. The reaction of tetrahydroderivatives **14** and **15** with polyfluoroaryl and alkyl sulfonylchlorides as well as with tosylchloride in dichloromethane in the presence of triethylamine produced tetrahydroberberrubine 9-*O*-sulfonates **11a**–**h** (44–84% yields) and 12-bromotetrahydroberberrubine 9-*O*-sulfonates **12a**–**h** (49–93% yields). New berberine type **10** derivatives were synthesized by the reaction of berberrubine hydrobromide **13** with different alkyl sulfochlorides. The reactions were carried out in dichloromethane in the presence of triethylamine for 5 h at room temperature. Sulfonates **10** were isolated by precipitation from the reaction mixtures.

The structures of the new compounds were confirmed by ^1^H-NMR,^13^C-NMR, IR and HRMS methods; the results are shown in the experimental section and the supplementary information.

### 2.2. Effects of the Berberine Sulfonates on Tdp1 Activity

An oligonucleotide real-time biosensor was used based on the ability of Tdp1 to remove fluorophore quenchers from the 3′-end of DNA, as previously described [31]. The hexadecameric oligonucleotide carried 5(6)-carboxyfluorescein (FAM) at the 5′-end and the fluorophore quencher BHQ1 (Black Hole Quencher-1) at the 3′-end. Tdp1 inhibitors prevent removal of fluorophore quenchers, thus reducing fluorescence intensity. The results of the Tdp1 assay for derivatives **10–12** and cytotoxic effects are shown in Table 1 and Table 2.

It is clear from the data in Table 1 that both alkyl sulfonates of berberine and tetrahydroberberine are not very active with the exception of the tetrahydroberberine derivatives containing both sufficiently long alkyl substituent and bromine in a *para*-position to the sulfonate substituent, **12c** and **12d**. The favorable substitution pattern of these derivatives is confirmed by the results of the tetrahydroberberine sulfonates with aromatic and polyfluoroaromatic substituents as shown in Table 2. Both sulfonates with *para*-toluenesulfonyl substituent (**11e** and **12e**) are inactive, but all three fluorinated sulfonates (**11f**, **11g**, **11h**) show good inhibitory activity with IC_50_ values of ~1 µM. Bromine substitution is not important for **12e–h** activity as comparison with their non-brominated analogues **11e–h** shows.

Based on the data shown in Table 1 and Table 2 it can be stated that some berberine derivatives inhibit Tdp1. It was found that the structure of the substituent in the sulfonate affects the inhibitory activity. Polyfluorinated arylsulfonates **11f–h** and **12f–h** exhibited inhibitory activity in the low micromolar range, while their non-fluorinated analogs (**11e**, **12e**) were inactive at these concentrations. In the series of alkylsulfonates, inhibitory activity was found only for compounds with a sufficiently long alkyl substituent (propyl-, butyl-) in the sulfonate group and in the presence of bromine substituent at position 12 (compounds **12c**,**d**). To explain the observed effects, a molecular modeling study was carried out. 

### 2.3. Molecular Modeling of the Berberine Derivatives

Twenty-three berberine derivatives were docked into the binding site of Tdp1 (PDB ID: 6DIE, resolution 1.78 Å) [32] with three water molecules (HOH814, 821 and 1078). It is established that keeping these crystalline water molecules improves the prediction quality of the docking scaffold (for further information see the Methodology section) [13]. The binding predictions of the scoring functions used are given in Table S1, all the ligands show reasonable scores. 

**Figure 3:**
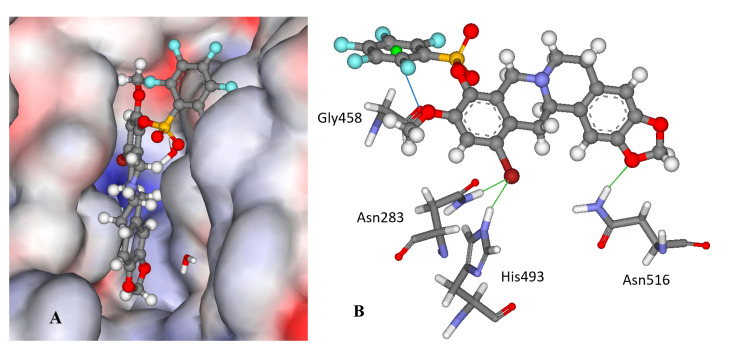
The docked configuration of **12f** in the binding site of Tdp1 as predicted by the ChemPLP scoring function. (**A**) The protein surface is rendered; blue depicts a hydrophilic region with a partial positive charge on the surface; red depicts hydrophobic region with a partial negative charge and grey shows neutral areas. The ligand occupies the catalytic pocket blocking access to it. (**B**) H-bonds are shown as green lines between **12f** and the amino acids Asn283, His493 and Asn516 side chains. A potential lone pair-π stacking interaction is shown as a blue line between the carboxylic backbone group in Gly458 and the centroid (green ball) of the fluorinated phenyl group (3.5 Å).

Compound **12f** is the ligand with the best IC_50_ value and according to the docking; **12f** fits neatly in the catalytic region as shown in Figure 3A. This region contains the catalytic histidine 263 and 493 amino acid residues thus the ligand blocs any activity of the enzyme. Indeed, **12f** forms a weak H-bond with the His493 imidazole site group via the bromine substituent as shown in Figure 3B as well as with Asn283’s amide side chain. Finally, the amide group of Asn516 forms an H-bond with one of the oxygen atoms in the 1.3-benzodioxole moiety of the ligand. It is worth mentioning that the methoxy group on **12f** can potentially form a weak H-bond with the thiol on Cys205, if the flexibility of the protein would be accounted for, and the same methoxy group has lipophilic contacts with Ile285 aliphatic side chain, stabilizing the binding mode. Interestingly, the modeling of the **11f** ligand, which does not contain a bromine group, but also with a good IC_50_ value, did not give consistent results across the scoring functions used, i.e., different conformations were predicted. This strongly indicates that the bromine group with its weak H-bonds to Asn283 and His493 is essential for anchoring the ligand in the catalytic site.

Interestingly, derivatives **11e** and **12e** are essentially inactive (IC_50_ >15 μM); they are structural analogues of the **11f**/**12f** pair with hydrogens on the phenyl ring as well as a *para* methyl substitution instead of fluorine groups. According to the modeling, the **11e**/**12e** pair has a different binding mode from **11f**/**12f** with the bromine moiety pointing into the aqueous phase for the former pair as shown in Figure S1 in the Supplementary Information. This indicates the importance of the fluorine substitution on the phenyl rings; the modeling suggests that the phenyl ring is leaning against the carboxyl moiety in the Gly458 backbone, which can form an interaction between the electron deficient fluoride substituted ring and the lone pairs of the oxygen atom in the carboxylic group as shown in Figure 3B. 

To investigate the binding stability of the ligands molecular dynamic (MD) runs were conducted using the docked conformations of **11f**, **12f** and **12e** for 10 ps at 1000 K. In general, the ligands are stable within the binding pocket and not ejected; the most stable intramolecular bond being between the fluorinated phenyl ring and the Gly458 carboxyl group for **12f**. In contrast, the phenyl group in **12e** is very mobile. The other H-bonding interactions predicted are often broken to be reestablished during the MD run.

### 2.4. Cytotoxicity

**Figure 4:**
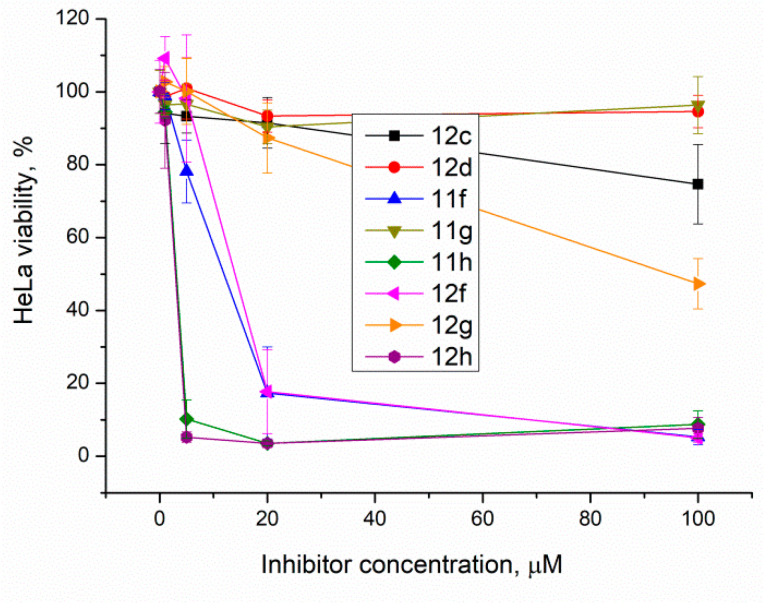
The berberine derivatives’ cytotoxicity according to the EZ4U test. Error bars show standard deviations.

Top1 poisons are used as anticancer drugs for the treatment for various oncological diseases [33,34,35]. Since Tdp1 is involved in the removal of DNA damage caused by Top1 poisons, the activity of Tdp1 can lead to the development of drug resistance [36]. Thus, it is believed that Tdp1 inhibition can enhance the efficacy of Top1 poisons [37]. Tdp1 inhibitors should have the lowest possible intrinsic toxicity to minimize potential side effects. Therefore, we studied the intrinsic cytotoxicity of the compounds against HeLa cells (cervical carcinoma). EZ4U cell proliferation and cytotoxicity assay results are shown in Figure 4 for the ligands with Tdp1 inhibitory activity.

The results show that 9-O-sulfonates of 12-bromotetrahydoberberine with aliphatic substituents **12c** and **12d**, (Table 1 and Figure 4, black and red traces) are non-toxic up to 100 μM. The toxicity of 9-O-sulfonates strongly depends on the structure of the polyfluorinated fragment. Compounds with a CF_3_-group in the *para*-position were the most toxic; CC_50_ values of 2.6 μM and 2.2 μM for **11h** and **12h,** respectively (Table 2 and Figure 4, green and brown traces). The replacement of the trifluoromethyl group with a fluorine atom in the *para*-position reduced toxicity with CC_50_ values of 10 μM for **11f** and **12f** (Table 2 and Figure 4, blue and magenta traces, respectively). The compounds with a hydrogen atom in the *para*-position were non-toxic (**11g**, Table 2 and Figure 4, violet trace) or moderately toxic (**12g**, Table 2 and Figure 4, orange trace). 

### 2.5. Sensitizing Effects

**Figure 5:**
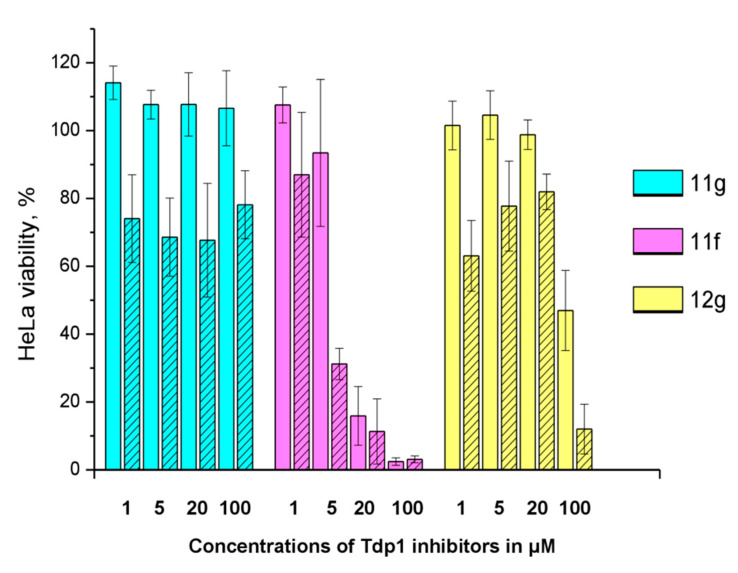
The influence of 2 μM topotecan on Tdp1 inhibitors’ cytotoxicity. The unshaded histogram bars denote cell viability in the presence of a Tdp1 inhibitor. The hatched histogram bars indicate cell viability in the presence of a combination of a Tdp1 inhibitor with 2 μM of topotecan. The concentration of the Tdp1 inhibitor in μM is given under each pair of bars. Error bars show standard deviations.

The sensitizing effect of the berberine inhibitors on topotecan’s cytotoxic potential was investigated. In order to determine the optimal concentration for the inhibitors to provide the maximum sensitizing effect, but remaining non-toxic, their concentrations were varied with topotecan concentration of 2 μM, its CC_50_ for HeLa cells. Topotecan significantly increased the cytotoxicity of compounds **11g**, **12g**, and **11f**, the reliability was confirmed by the Mann–Whitney U-test, *p* = 0.05 and the results are shown in Figure 5. The original data are given in Table S2.

**11g** is non-toxic in the concentration range used; 100% of living cells with a 100 μM maximum concentration. In the presence of topotecan, a significant decrease in cell survival is observed (~30%) at all concentrations. Compound **12g** has a low toxicity potential of 95 μM (CC_50_). Topotecan weakly, but significantly reduces this value to 62 μM. Compound **11f** is inherently toxic, and 90% of the cells die at 20 μM. At lower concentrations, the effect of topotecan is significant. CC_50_ value for **11f** decreases three fold, from 11 to 3.7 μM, in the presence of topotecan. For other ligands (**11h**, **12c**, **d**, **f**, **h**), the effect of topotecan was negligible or unreliable. 

**Figure 6:**
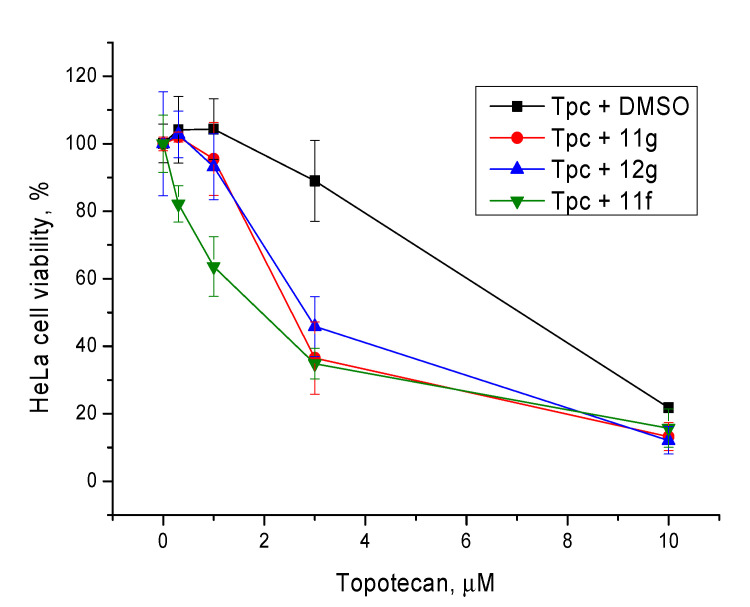
The influence of the Tdp1 inhibitors at 5 µM on topotecan cytotoxicity. Error bars show standard deviations.

Non-toxic concentrations of the berberine derivatives (5 μM) were then tested at different concentrations of topotecan. The most toxic compound **11f** caused 20% cell death at this concentration; the rest of the compounds were not toxic. In general, our Tdp1 inhibitors doubled the cytotoxic potential of topotecan as can be seen in Figure 6 and Table 3.

**Figure 7:**
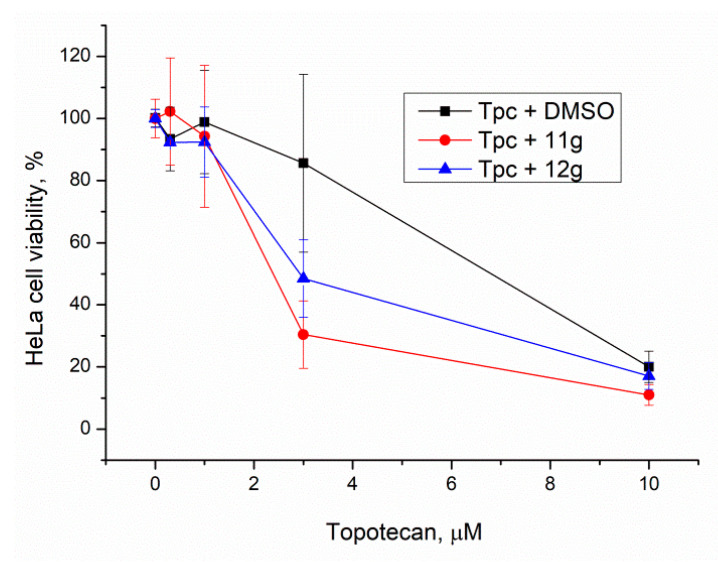
Influence of Tdp1 inhibitors at 20 μM on topotecan cytotoxicity. Error bars show standard deviations.

For comparison, the concentration of the inhibitors was increased to 20 μM. Compound **11f** was not used due to its high toxicity. Again, compounds **12g** and **11g** had a significant effect, *p* < 0.05, confirmed by the Mann–Whitney U-test (Figure 7 and Table 3). It is interesting to note that the sensitizing effect of Tdp1 inhibitors was practically the same at 5 and 20 μM.

Derivatives **11g** and **12g** can be considered to be the lead compounds since they are, unlike **11f**, non-toxic (**11g**) or moderately toxic (**12g**) and have a pronounced sensitizing effect on topotecan.

### 2.6. Chemical Space

The calculated molecular descriptors MW (molecular weight), log *P* (water-octanol partition coefficient), HD (hydrogen bond donors), HA (hydrogen bond acceptors), PSA (polar surface area) and RB (rotatable bonds)) are given in Table S3. The MW of the ligands lies between 325.4 and 684.4 g mol^−1^ and falls into drug-like and Known Drug Space (KDS) regions. Log *P* spans from 1.6 to 5.4, i.e., over the three defined volumes in chemical space, with most in lead-like chemical space and only one ligand **12h** in KDS. The HD, HA, RB and PSA values are within the lead- and drug-like definitions (for the definition of lead-like, drug-like and Known Drug Space regions see ref. [38] and Table S4). 

The Known Drug Indexes (KDIs) for the ligands were calculated to gauge the balance of the molecular descriptors (MW, log P, HD, HA, PSA and RB). This method is based on the analysis of drugs in clinical use, i.e., the statistical distribution of each descriptor is fitted to a Gaussian function and normalized to 1 resulting in a weighted index. Both the summation of the indexes (KDI_2a_—Equation (1)) and multiplication (KDI_2b_—Equation (2)) methods were used [39]. The numerical results are given in Table S3 in the supplementary information.

KDI_2a_ = I_MW_ + I_log P_ + I_HD_+ I_HA_ + I_RB_ + I_PSA_ (1)

KDI_2b_ = I_MW_ × I_log P_ × I_HD_× I_HA_ × I_RB_ × I_PSA_ (2)

The KDI_2a_ values range from 4.35 to 5.70 with a theoretical maximum of 6 and the average of 4.08 for known drugs. KDI_2b_ range from 0.06 to 0.73, with a theoretical maximum of 1 and with KDS average of 0.18. The berberine ligands can be considered reasonably well balanced in terms of their molecular descriptors and therefore biocompatible. The most active compound **12f**, has a KDI_2a_ value of 4.58 and KDI_2b_ of 0.12; the relatively low KDI_2b_ value can be explained by its high MW, which is compensated by the favorable values for the other descriptors resulting in a good KDI_2a_ value. The KDI_2b_ index is sensitive to any outliers since multiplication of small numbers leads to small numbers.

## 3. Materials and Methods

### 3.1. Chemistry 

Berberine chloride was purchased from Tokyo Chemical Industry Co., Ltd (Tokyo, Japan). Methanesulfochloride and ethanesulfochloride were purchased from Acros Organics (Belgium), 1-propanesulfochloride and 1-buthanesulfochloride were purchased from Alfa Aesar (Karlsruhe, Germany). 48% aqueous HBr solution was purchased from Acros Organics (the Netherlands). All solvents used in the reactions were purified and dried. Bromine, sodium borohydride and triethylamine were purchased from Sigma-Aldrich. Column chromatography was carried out on neutral alumina LL40/250. All reactions were monitored by TLC analysis using Merck Aluminium oxide 60 F_254_ plastic sheets (Darmstadt, Germany), eluent CH_2_Cl_2_-MeOH.

The ^1^H and ^13^C NMR spectra were recorded on a Bruker AM-400 spectrometer (400.13 and 100.61 MHz) for 5–10% solutions of the compounds in CDCl_3_ or DMSO-d_6_ using the signal of the solvent CDCl_3_ as the standard (δ 7.24 for 1H and δ 76.90 for 13C). The IR spectra were measured on a Vector 22 FTIR spectrometer in KBr pellets. High-resolution electrospray ionization (HRESI) mass spectra were carried out using a time-of-flight high-resolution mass spectrometer micrOTOF-Q (Bruker Daltonics, Germany) with an Agilent 1200 liquid chromatograph (Agilent Technologies, USA/Germany). Positive ion scanning in the range *m*/*z* = 100–3000. Drying gas (nitrogen) flow rate of 4 L/min; temperature—190 ℃; sprayer pressure—1.0 bar.

The spectroscopic and analytical measurements were carried out at the Multi-access Chemical Service Center of the Siberian Branch of the Russian Academy of Sciences.

#### 3.1.1. General Synthesis of Compounds **11a–g** and **12a–g**

Desired compounds **11a–d**, **f**, **g** and **12a–g** were synthesized according to the general procedure described previously [27], compound **11e** was also synthesized according to the procedure described previously [30]. 

#### 3.1.2. General Procedure for the Synthesis of Sulfonates **10a–d**

2.5 mmol of triethylamine was added to the suspension of 1 mmol of berberrubine hydrobromide in 25 mL of methylene chloride. 1.5 mmol of sulfochloride in 3 mL of methylene chloride was added under stirring at room temperature. After 5 h the precipitate was filtered, washed with methylene chloride and the pure product **10a–d** was isolated. 

#### 3.1.3. 10-Methoxy-9-methanesulfonyloxy-2,3-methylenedioxyprotoberberine chloride (**10a**) 

##### Yield: 7%

Spectra NMR ^1^H (400 MHz, DMSO-d_6_, δ, ppm, *J*/Hz): 3.22 (2H, *t*, *J* = 6.07 Hz, H-5), 3.73 (3H, s, SCH_3_), 4.12 (3H, s, OCH_3_), 5.00 (2H, *t*, *J* = 5.72 Hz, H-6), 6.19 (2H, s, OCH_2_O), 7.11 (1H, s, H-4), 7.82 (1H, s, H-1), 8.27 (1H, *d*, *J* = 9.06 Hz, H-12), 8.34 (1H, *d*, *J* = 9.06 Hz, H-11), 9.08 (1H, s, H-13), 9.72 (1H, s, H-8). Spectra NMR ^13^C (100 MHz, DMSO-d_6_, δ, ppm): 26.18 (C-5), 40.02 (SO_2_*CH_3_*), 55.70 (C-6), 57.44 (OCH_3_), 102.23 (OCH_2_O), 105.60 (C-1), 108.49 (C-4), 120.20 (C-13b), 120.86 (C-13), 121.79 (C-8a), 126.47 (C-12), 128.03 (C-11), 131.05 (C-4a), 131.44 (C-12a), 133.31 (C-13a), 138.59 (C-9), 144.04 (C-8), 147.77 (C-2), 150.17 (C-3), 151.89 (C-10). Spectra IR (cm^−1^): 804.21, 925.71, 1033.71, 1097.35, 1224.63, 1263.20, 1365.42, 1506.20, 1610.34, 1623.84, 3039.40, 3320.97, 3635.33. MS (ESI): m/z (M^+^) calcd for C_20_H_18_NO_6_S^+^, 400.085 found: 400.081.

#### 3.1.4. 9-Ethanesulfonyloxy-10-methoxy-2,3-methylenedioxyprotoberberine chloride (**10b**) 

##### Yield: 25%

Spectra NMR ^1^H (400 MHz, DMSO-d_6_, δ, ppm, *J*/Hz): 1.52 (3H, *t*, *J* = 7.32 Hz, CH_2_*CH_3_*), 3.22 (2H, *t*, *J* = 6.05 Hz, H-5), 3.89 (2H, q, J_1_ = 3.50 Hz, J_2_ = 10.96 Hz, *CH_2_*CH_3_), 4.12 (3H, s, OCH_3_), 5.01 (2H, t, *J* = 5.91 Hz, H-6), 6.18 (2H, s, OCH_2_O), 7.11 (1H, s, H-4), 7.82 (1H, s, H–1), 8.27 (1H, *d*, *J* = 9.48 Hz, H-12), 8.34 (1H, *d*, *J* = 9.48 Hz, H-11), 9.10 (1H, s, H-13), 9.64 (1H, s, H–8). Spectra NMR ^13^C (100 MHz, DMSO-d_6_, *δ*, ppm): 8.18 (CH_2_*CH_3_*), 26.19 (C-5), 47.00 (SCH_2_), 55.83 (C–6), 57.44 (OCH_3_), 102.20 (OCH_2_O), 105.61 (C-1), 108.45 (C–4), 120.18 (C-13b), 120.90 (C-13), 121.98 (C-8a), 126.42 (C-12), 128.02 (C-11), 131.02 (C-4a), 131.27 (C-12a), 133.36 (C-13a), 138.63 (C-9), 143.91 (C-8), 147.75 (C-2), 150.17 (C-3), 151.71 (C-10). Spectra IR (cm^−1^): 806.14, 923.78, 1033.71, 1097.35, 1164.85, 1222.70, 1278.2063, 1363.49, 1506.20, 1608.42, 1621.92, 3023.98, 3423.19, 3629.54. MS (ESI): *m*/*z* (M^+^) calcd for C_21_H_20_NO_6_S^+^, 414.101 found: 414.096.

#### 3.1.5. 10-Methoxy-2,3-methylenedioxy-9-((propane-1-sulfonyl)oxy)protoberberine chloride (**10c**) 

##### Yield: 34%

Spectra NMR ^1^H (400 MHz, DMSO-d_6_, δ, ppm, J/Hz): 1.11 (3H, t, *J* = 7.45 Hz, CH_2_*CH_3_*), 1.94–2.03 (2H, m, *CH_2_*CH_3_), 3.22 (2H, *t*, *J* = 5.72 Hz, H-5), 3.87 (2H, t, *J* = 7.58 Hz, S*CH_2_*CH_2_), 4.12 (3H, s, OCH_3_), 5.01 (2H, *t*, *J* = 6.02 Hz, H-6), 6.19 (2H, s, OCH_2_O), 7.11 (1H, s, H-4), 7.82 (1H, s, H-1), 8.27 (1H, *d*, *J* = 9.33 Hz, H-12), 8.34 (1H, d, J = 9.33 Hz, H-11), 9.10 (1H, s, H-13), 9.64 (1H, s, H-8). Spectra NMR ^13^C (100 MHz, DMSO-d_6_, δ, ppm): 12.49 (CH_2_*CH_3_*), 17.17 (CH_2_*CH_2_*CH_3_), 26.16 (C-5), 53.27 (SCH_2_), 55.80 (C-6), 57.46 (OCH_3_), 102.18 (OCH_2_O), 105.60 (C-1), 108.42 (C-4), 120.14 (C-13b), 120.86 (C-13), 121.93 (C-8a), 126.37 (C-12), 127.98 (C-11), 130.96 (C-4a), 131.23 (C-12a), 133.33 (C-13a), 138.55 (C-9), 143.91 (C-8), 147.70 (C-2), 150.12 (C-3), 151.69 (C-10). Spectra IR (cm^−1^): 817.71, 925.71, 1037.56, 1095.42, 1162.92, 1222.70, 1263.20, 1363.49, 1506.20, 1610.34, 1621.92, 3023.98, 3427.04. MS (ESI): *m*/*z* (M^+^) calcd for C_22_H_22_NO_6_S^+^, 428.116 found: 428.121.

#### 3.1.6. 9-(Butane-1-sulfonyl)oxy-10-methoxy-2,3-methylenedioxy protoberberine chloride (**10d**) 

##### Yield: 50%

Spectra NMR ^1^H (400 MHz, DMSO-d_6_, δ, ppm, *J*/Hz): 0.97 (3H, t, *J* = 7.34 Hz, CH_2_*CH_3_*), 1.48–1.58 (2H, m, *CH_2_*CH_3_), 1.89–1.97 (2H, m, SCH_2_*CH_2_*), 3.22 (2H, t, *J* = 5.96 Hz, H-5), 3.91 (2H, t, *J* = 7.68 Hz, SCH_2_), 4.11 (3H, s, OCH_3_), 5.02 (2H, t, *J* = 6.04 Hz), 6.18 (2H, c, OCH_2_O), 7.10 (1H, s, H-4), 7.82 (1H, s, H-1), 8.27 (1H, *d*, *J* = 9.21 Hz, H-12), 8.34 (1H, *d*, *J* = 9.21 Hz, H-11), 9.13 (1H, s, H-13), 9.67 (1H, s, H-8). Spectra NMR ^13^C (100 MHz, DMSO-d_6_, δ, ppm): 13.44 (*CH_3_*CH_2_), 20.74 (*CH_3_*CH_2_), 25.29 (SCH_2_*CH_2_*), 26.19 (C-5), 51.75 (SCH_2_), 55.80 (C-6), 57.46 (OCH_3_), 102.20 (OCH_2_O), 105.62 (C-1), 108.45 (C-4), 120.19 (C-13b), 120.91 (C-13), 121.98 (C-8a), 126.42 (C-12), 128.01 (C-11), 131.02 (C-4a), 131.30 (C-12a), 133.36 (C-13a), 138.62 (C-9), 143.97 (C-8), 147.75 (C-2), 150.16 (C-3), 151.72 (C-10). Spectra IR (cm^−1^): 811.92, 925.71, 1035.63, 1099.28, 1222.70, 1265.13, 1365.42, 1504.27, 1610.34, 1619.99, 2960.33, 3425.11. MS (ESI): *m*/*z* (M^+^) calcd for C_23_H_24_NO_6_S^+^, 442.132 found: 442.133.

### 3.2. Biology

#### 3.2.1. Detection of Tdp1 Activity

The methodology has been reported in our previous work [30] and consists of fluorescence intensity measurement in a reaction of quencher removal from a fluorophore quencher-coupled DNA oligonucleotide catalyzed by Tdp1. The reaction was carried out at different concentrations of inhibitors (the control samples contained 1% of DMSO, Sigma, St. Louis, MO, USA). The reaction mixtures contained Tdp1 buffer (50 mM Tris-HCl pH 8.0, 50 mM NaCl, and 7 mM β-mercaptoethanol), 50 nM biosensor, and an inhibitor being tested. Purified Tdp1 (1.5 nM) triggered the reaction. The biosensor (5′-[FAM] AAC GTC AGGGTC TTC C [BHQ]-3′) was synthesized in the Laboratory of Biomedical Chemistry at the Institute of Chemical Biology and Fundamental Medicine (Novosibirsk, Russia).

The reactions were incubated on a POLARstar OPTIMA fluorimeter (BMG LABTECH, GmbH, Ortenberg, Germany) to measure fluorescence every 55 s (ex. 485/em. 520 nm) during the linear phase (here, data from minute 0 to minute 8). The values of IC_50_ were determined using a six-point concentration response curve in minimum three independent experiments and were calculated using MARS Data Analysis 2.0 (BMG LABTECH, GmbH, Ortenberg, Germany).

#### 3.2.2. Cytotoxicity Assays 

Cytotoxicity of the compounds to HeLa (human cervical cancer) cell line was examined using the EZ4U Cell Proliferation and Cytotoxicity Assay (Biomedica, Vienna, Austria), according to the manufacturer’s protocols. The cells were grown in Iscove’s modified Dulbecco’s medium (IMDM) with 40 μg/mL gentamicin, 50 IU/mL penicillin, 50 μg/mL streptomycin (MP Biomedicals, Santa Ana, CA, USA), and 10% of fetal bovine serum (Biolot, St. Petersburg, Russia) in a 5% CO_2_ atmosphere. After formation of a 30–50%-monolayer, the tested compounds were added to the medium. The volume of the added reagents was 1/100 of the total volume of the culture medium, and the amount of DMSO (Sigma, St. Louis, MO, USA) was 1% of the final volume. Control cells were grown in the presence of 1% DMSO. The cell culture was monitored for 3 days. To assess the influence of the inhibitors on the cytotoxic effect of topotecan (ACTAVIS GROUP PTC ehf., Bucharest, Romania), 50% cytotoxic concentrations of topotecan and of each inhibitor were determined to attain a defined single-agent effect. Then, minimum two independent tests were performed with each inhibitor in combination with topotecan. When using a combination of drugs, Tdp1 inhibitors were first added, then topotecan was added immediately (within 10–15 min).

### 3.3. Molecular Modeling

The compounds were docked against the crystal structure of TDP1 Tdp1 (PDB ID: 6DIE, resolution 1.78 Å) [32] which was obtained from the Protein Data Bank (PDB) [40,41]. The Scigress version FJ 2.6 program [42] was used to prepare the crystal structure for docking, i.e., the hydrogen atoms were added, the co-crystallized ligand benzene-1,2,4-tricarboxylic acid was removed as well as crystallographic water molecules except HOH 814, 821 and 1078. The waters were set on toggle—bound or displaced by the ligand during docking—and spin—automatic optimization of the orientation of the hydrogen atoms. The Scigress software suite was also used to build the inhibitors and the MM2 [43] force field was used to optimize the structures. Furthermore, Scigress was used for the 10 ps MD runs at 1000 K; the MM2 force filed was used, 5 Å radius was defined around the ligand and allowed to be flexible whereas the rest of the protein structure was held ridged (locked). First the binding pocket with the ligand was structurally optimized followed by the MD run. The docking center was defined as the position of a carbon on the ring of the co-crystallized benzene-1, 2, 4-tricarboxylic acid (*x* = −6.052, *y* = −14.428, *z* = 33.998) with 10 Å radius. Fifty docking runs were allowed for each ligand with default search efficiency (100%). The basic amino acids lysine and arginine were defined as protonated. Furthermore, aspartic and glutamic acids were assumed deprotonated. The GoldScore (GS) [44] and ChemScore (CS) [45,46] ChemPLP (Piecewise Linear Potential) [47] and ASP (Astex Statistical Potential) [48] scoring functions were implemented to predict the binding modes and relative energies of the ligands using the GOLD v5.4.1 software suite.

The QikProp 3.2 [49] software package was used to calculate the molecular descriptors of the molecules. The reliability of it QikProp established for the calculated descriptors. [50] The Known Drug Indexes (KDI) were calculated from the molecular descriptors as described by Eurtivong and Reynisson [40]. Five of the compounds (**9** and **10a–d**) carry a positive charge and QikProp does not compute charged molecules. The MW, Log P, HD and HA were derived using the Scigress version FJ 2.6 program [42] software, PSA and RB are not available in this software suite.

## 4. Conclusions

The berberine and tetrahydroberberine sulfonates and their brominated analogues were tested to evaluate their Tdp1 inhibitory activity. To our knowledge, this is the first report that documents berberine-based compounds as inhibitors of this enzyme. The IC_50_ values are in the 0.53 to 4 μM range. Of the alkyl sulfonates, only derivatives with bromine substitution on site 12 of tetrahydroberberrubine are active. While both tetrahydroberberrubine and 12-bromtetrahydroberberrubine derivatives containing polyfluoroaromatic substituents all have inhibitory activity with IC_50_ values of ~1 μM. According to the inhibitory activity, toxicity data and ability to sensitize topotecan against the HeLa cancer cell line the two most promising derivatives were identified—**11g** and **12g**. These results indicate that sulfonates of tetrahydroberberine have potential to be developed as new agents for anticancer therapy due to their inhibitory activity and lack of toxicity.

## Figures and Tables

**Table 1:** Tdp1 inhibiting activity (IC_50_—half maximal inhibitory concentration) of sulfonate berberine derivatives with aliphatic substituents and their cytotoxicity (CC_50_—half maximal cytotoxic concentration) in HeLa cells. Furamidine was used as a positive control (IC_50_ = 1.2 ± 0.3 µM).

Code, Structure	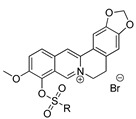 **10a–d**		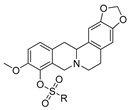 **11a–d**		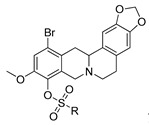 **12a–d**	
R	IC_50_, µM	CC_50_, µM	IC_50_, µM	CC_50_, µM	IC_50_, µM	CC_50_, µM
**a**; Me	>15	ND *	>15	ND	>15	ND
**b**; Et	>15	ND	>15	ND	>15	ND
**c**; Pr	>15	ND	>15	ND	2.9 ± 1.3	>100
**d**; Bu	>15	ND	>15	ND	4 ± 1.0	>100

* ND—not determined.

**Table 2:** Tdp 1 inhibiting activity (IC_50_) of sulfonate berberine derivatives with polyfluoroaromatic substituents and their cytotoxicity (CC_50_) in HeLa cells. Furamidine was used as a positive control (IC_50_ = 1.2 ± 0.3 µM).

Code, structure	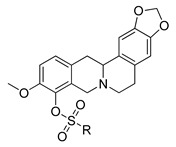 **11e–h**		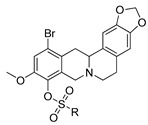 **12e–h**	
**R**	IC_50_, µM	CC_50_, µM	IC_50_, µM	CC_50_, µM
**e;** 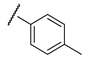	>15	ND	>15	ND
**f;** 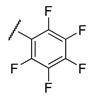	1.0 ± 0.20	11 ± 2.0	0.53 ± 0.01	9.9 ± 4.5
**g;** 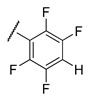	1.05 ± 0.05	>100	1.3 ± 0.30	95 ± 5.0
**h;** 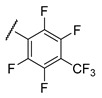	0.9 ± 0.20	2.6 ± 0.1	1.4 ± 0.30	2.2 ± 1.5

**Table 3:** The influence of the Tdp1 inhibitors at 5 µM on the cytotoxic potential of topotecan (Tpc).

Compounds	CC_50_, μM–5 μM Tdp1 inhibitor	CC_50_, μM–20 μM Tdp1 inhibitor
Tpc	6.8 ± 1.1	
Tpc + **11g**	3.5 ± 0.6	2.3 ± 0.5
Tpc + **12g**	2.9 ± 0.4	3.3 ± 1.1
Tpc + **11f**	1.7 ± 0.3	not determined

## Supplementary Materials

Supplementary Materials can be found at https://www.mdpi.com/1422-0067/21/19/7162/s1.

## Abbreviations

Tdp1	Tyrosyl-DNA phosphodiesterase 1
PARP	Poly (ADP-ribose) polymerase
