# Supplementary material for: The First Berberine-Based Inhibitors of Tyrosyl-DNA Phosphodiesterase 1 (Tdp1), an Important DNA Repair Enzyme
Source: Int J Mol Sci. 2020 Sep 28;21(19):7162. doi: 10.3390/ijms21197162 (PMC7582571; doi:10.3390/ijms21197162)
Supplement: Supplementary file 1 [file ijms-21-07162-s001.pdf]

SUPPLEMENTARY MATERIALS

The first berberine-based inhibitors of tyrosyl-DNA phosphodiesterase 1 (Tdp1), an important DNA repair enzyme

Elizaveta D. Gladkova, Ivan V. Nechepurenko, Roman A. Bredikhin, Arina A. Chepanova, Alexandra L. Zakharenko, Olga A. Luzina, Ekaterina S. Ilina, Nadezhda S. Dyrkheeva, Evgeniya M. Mamontova, Rashid O. Anarbaev, Jóhannes Reynisson, Konstantin P. Volcho, Nariman F. Salakhutdinov and Olga I. Lavrik

NMR ^{1}H and ^{13}C spectra of the compounds **10a - 10d** (solvent – DMSO-d_6).

Table S1. The binding scores for the scoring functions used.

Table S2. The molecular descriptors and their corresponding Known Drug Indexes 2a and 2b (KDI2a/2b).

Table S3. Definition of lead-like, drug-like and Known drug space (KDS) in terms of molecular descriptors. The values given are the maxima for each descriptor for the volumes of chemical space used.

Compound **10a** (^{1}**H NMR)**

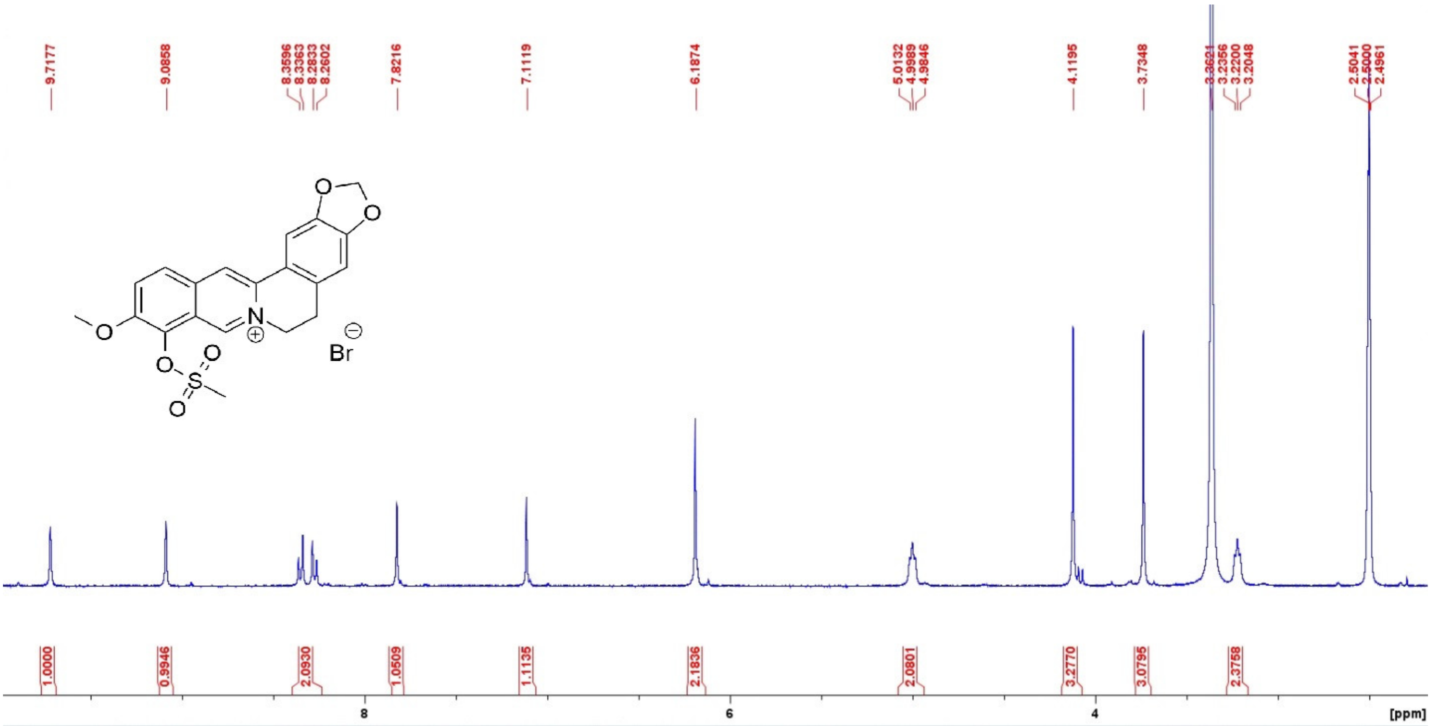

Compound **10a** (^{13}**C NMR)**

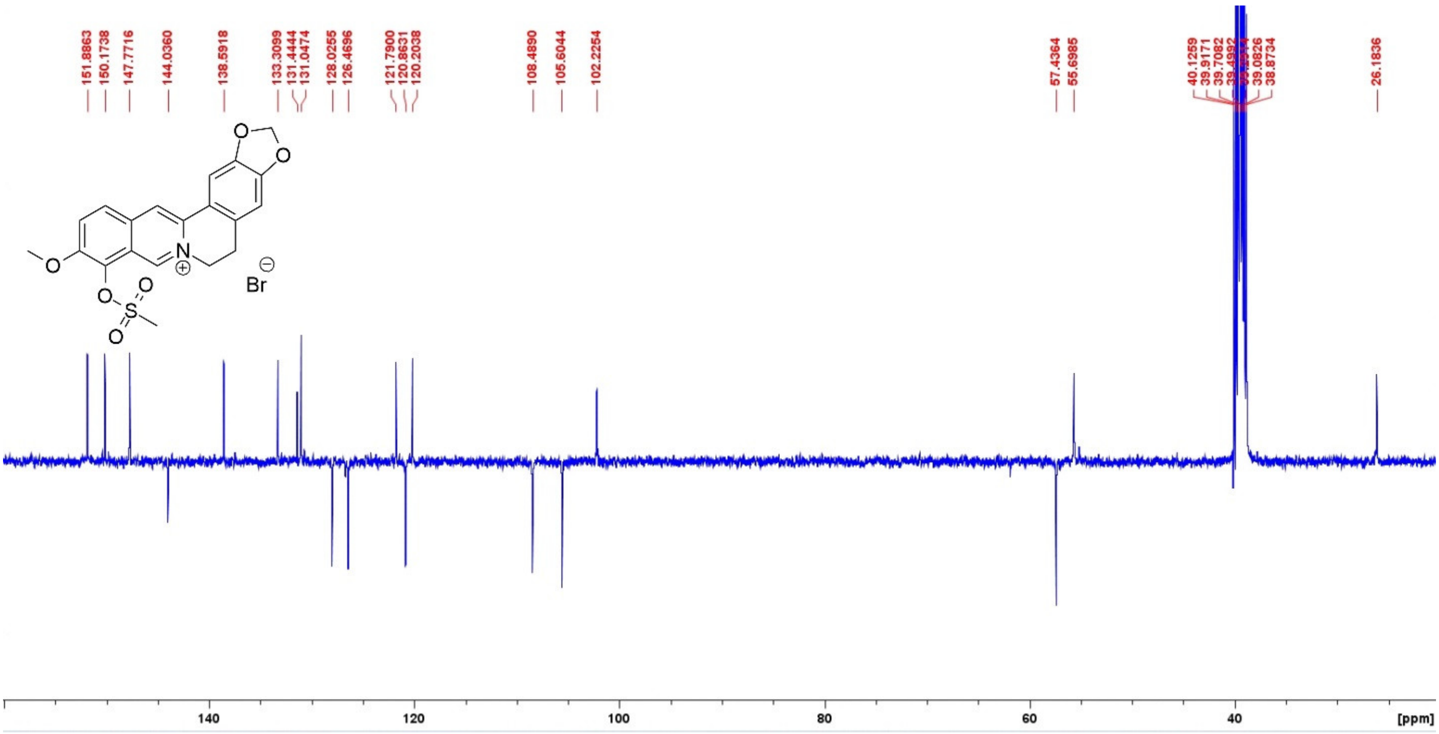

Compound **10b** (^{1}H NMR)

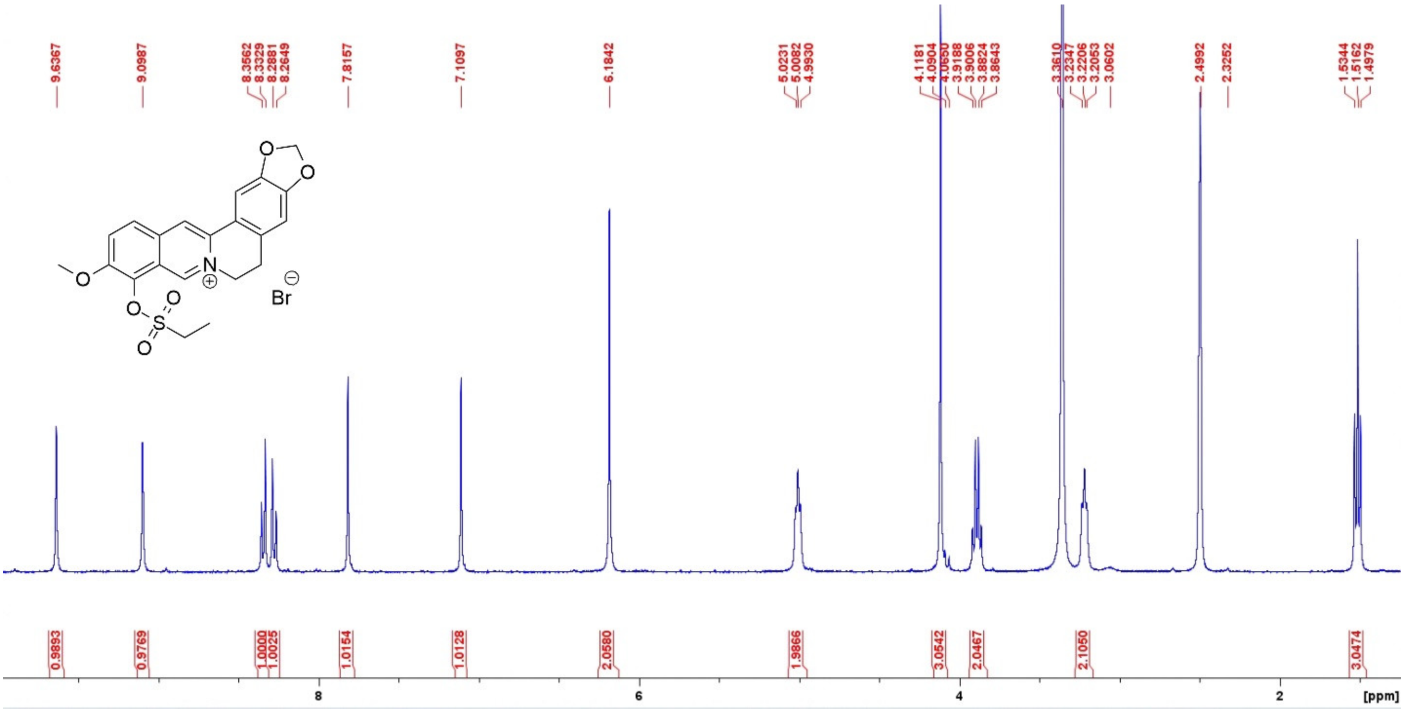

Compound **10b** (^{13}C NMR)

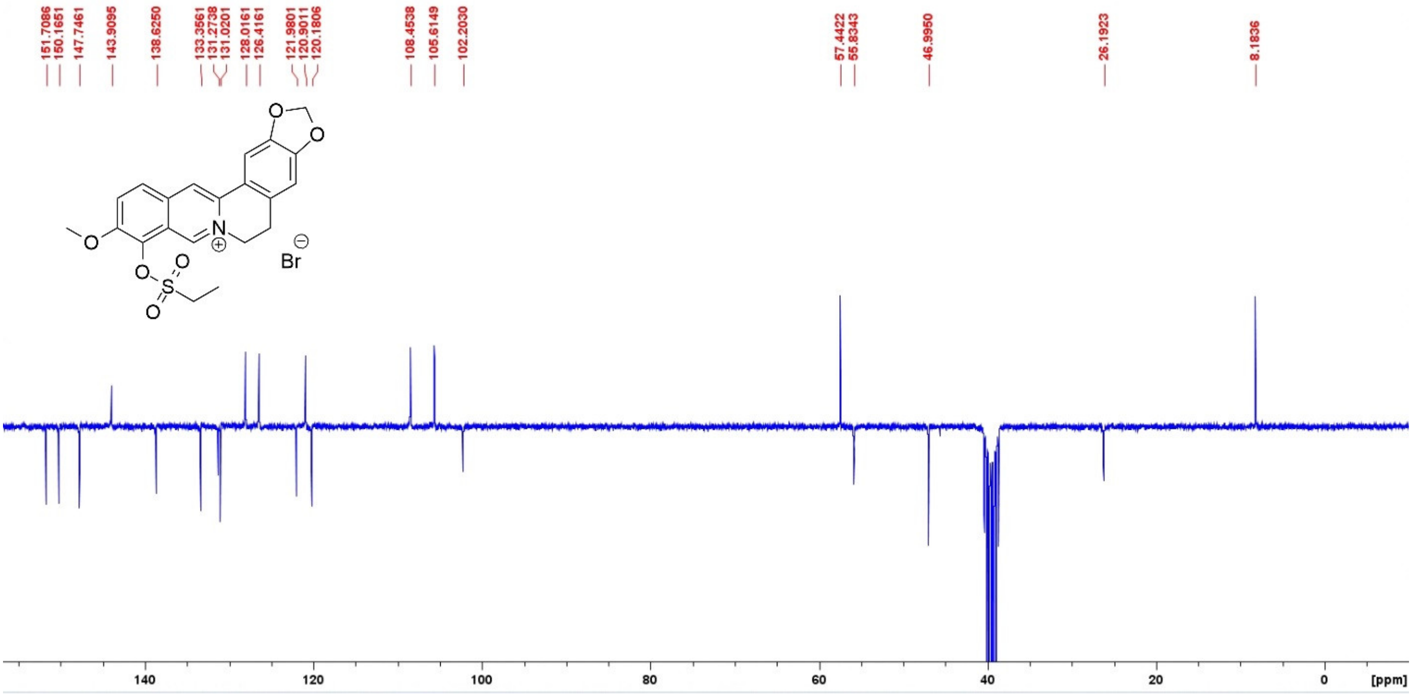

Compound **10c** (^{1}H NMR)

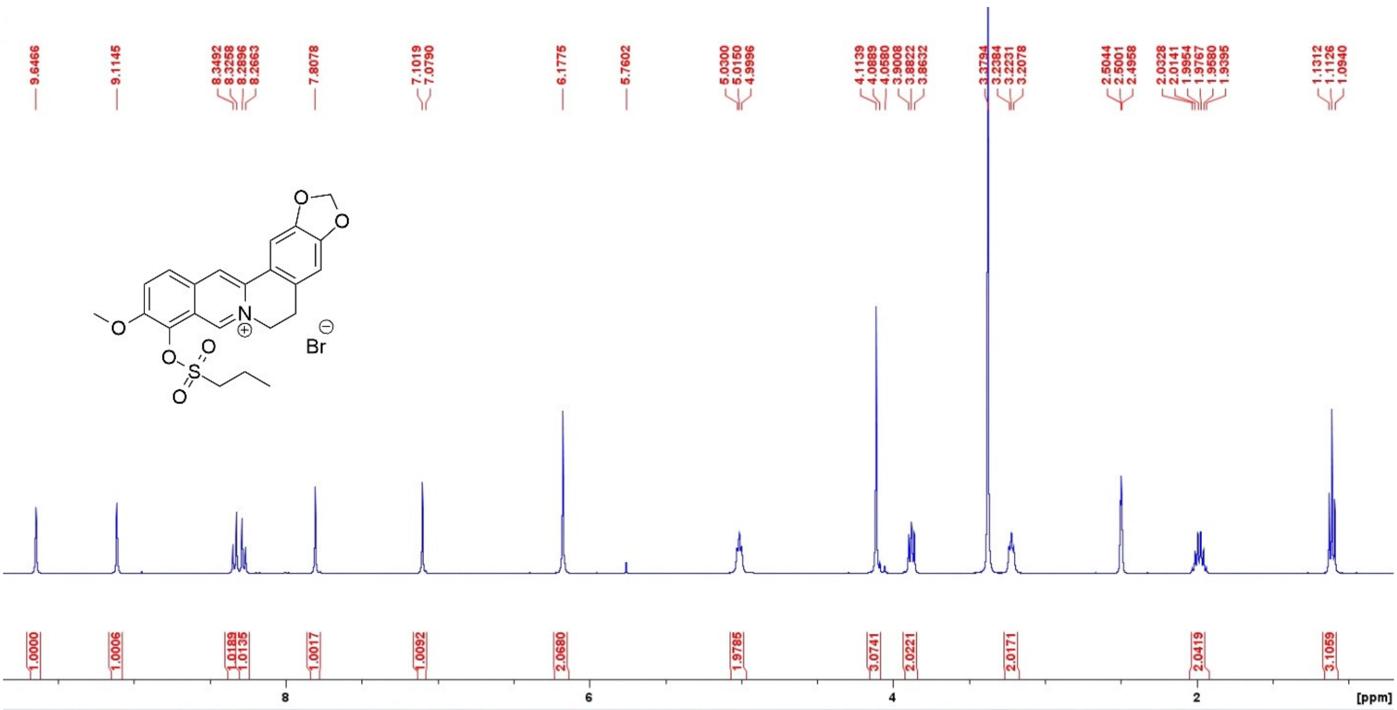

Compound **10c** (^{13}C NMR)

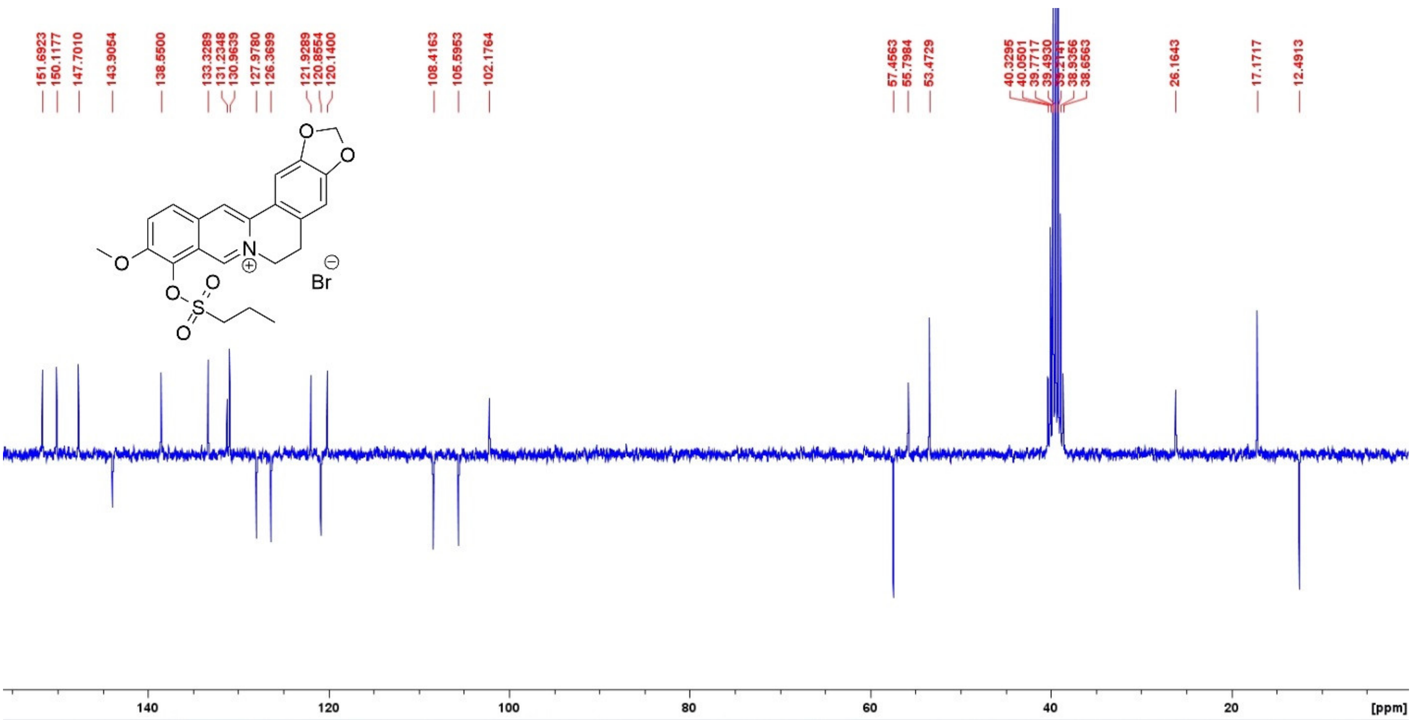

Compound **10d** (^{1}H NMR)

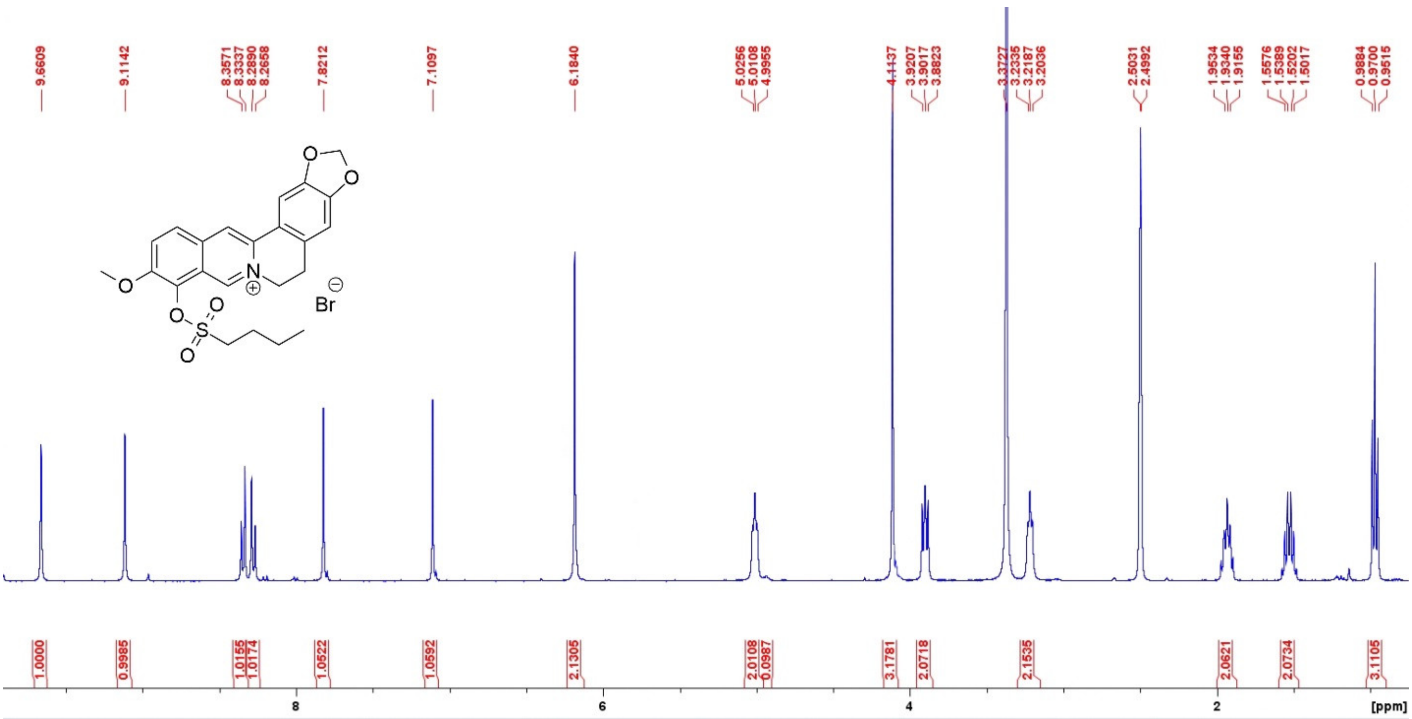

Compound **10d** (^{13}C NMR)

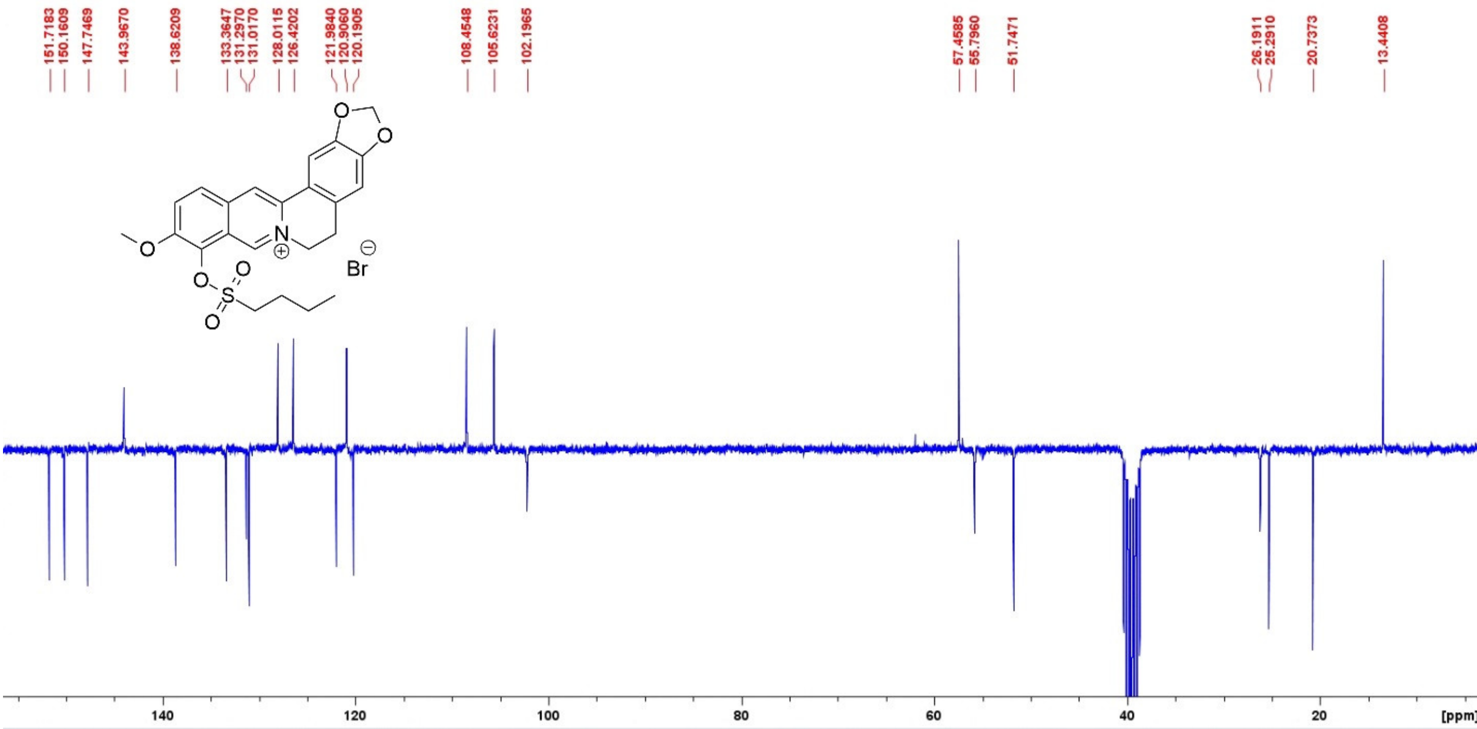

Table S1. The binding scores for the scoring functions used.

Ligand	ASP	ChemScore	GoldScore	ChemPLP	IC_{50} (μM)
**9**	31.0	23.4	48.8	55.9	>15
**10a**	33.4	27.6	59.6	62.0	>15
**10b**	34.9	28.1	60.9	63.3	>15
**10c**	33.8	27.9	66.2	62.8	>15
**10d**	34.4	29.0	59.6	72.6	>15
**11a**	34.3	26.5	52.0	55.0	>15
**11b**	31.4	27.9	58.8	59.6	>15
**11c**	32.6	24.9	55.4	59.7	>15
**11d**	33.1	27.0	57.4	63.9	>15
**11e**	38.1	31.6	67.9	63.1	>15
**11f**	39.2	24.1	58.8	65.7	0.98±0.20
**11g**	38.2	25.3	62.7	64.8	1.045±0.05
**11h**	42.5	26.5	67.4	70.2	0.93±0.22
**12a**	34.0	25.0	56.6	61.0	>15
**12b**	34.8	28.4	60.4	63.1	>15
**12c**	33.3	28.0	61.3	60.8	2.86±1.25
**12d**	34.6	27.1	58.2	59.8	4.06±1.05
**12e**	39.0	31.6	67.9	64.3	>15
**12f**	38.4	26.3	59.4	60.9	0.53±0.01
**12g**	38.3	27.4	59.0	62.3	1.33±0.28
**12h**	39.1	25.9	58.5	69.1	1.38±0.30

Table S2. The molecular descriptors and their corresponding Known Drug Indexes 2a and 2b (KDI2a/2b).

**Name**	**MW**	**Log P**	**HA**	**HD**	**PSA**	**Rot.Bond.**	**KDI2a**	**KDI2b**
**9**[a]	336.4	2.3	4	0	x	x	x	x
**10a**[a]	400.4	1.6	6	0	x	x	x	x
**10b**[a]	414.5	1.9	6	0	x	x	x	x
**10c**[a]	428.5	2.4	6	0	x	x	x	x
**10d**[a]	442.5	2.8	6	0	x	x	x	x
**11a**	403.4	1.7	8.75	0	67.1	3	5.36	0.48
**11b**	417.5	2.0	8.75	0	67.4	4	5.40	0.51

**11c**	431.5	2.1	8.75	0	72.9	5	5.39	0.50
**11d**	445.5	2.8	8.75	0	71.8	6	5.34	0.47
**11e**	479.5	3.4	8.75	0	77.0	4	5.27	0.43
**11f**	555.5	4.2	8.75	0	71.1	4	4.91	0.26
**11g**	537.5	3.9	8.75	0	71.1	4	5.00	0.30
**11h**	605.5	4.9	8.75	0	69.1	4	4.62	0.15
**12a**	482.3	2.3	8.75	0	66.1	3	5.21	0.41
**12b**	496.4	2.5	8.75	0	73.4	4	5.22	0.41
**12c**	510.4	2.6	8.75	0	72.6	5	5.16	0.38
**12d**	524.4	3.3	8.75	0	71.4	6	5.06	0.33
**12e**	558.4	3.9	8.75	0	75.0	4	4.93	0.26
**12f**	634.4	4.6	8.75	0	71.6	4	4.58	0.12
**12g**	616.4	4.4	8.75	0	72.1	4	4.67	0.15
**12h**	684.4	5.4	8.75	0	70.9	4	4.35	0.06

[a] Values generated using Scigress, x – no values

Table S3. Definition of lead-like, drug-like and Known drug space (KDS) in terms of molecular descriptors. The values given are the maxima for each descriptor for the volumes of chemical space used.

	**Lead-like Space**	**Drug-like Space**	**Known Drug Space**
Molecular weight (g mol^{-1})	300	500	800
Lipophilicity (Log P)	3	5	6.5
Hydrogen bond donors (HD)	3	5	7
Hydrogen bond acceptors (HA)	3	10	15
Polar surface area (Å^2) (PSA)	60	140	180
Rotatable bonds (RB)	3	10	17
